# Diagnostic value of contrast-enhanced ultrasonography in the preoperative evaluation of lymph node metastasis in papillary thyroid carcinoma: a single-center retrospective study

**DOI:** 10.1186/s12893-023-02199-w

**Published:** 2023-10-24

**Authors:** Zhixin Yang, Xiaofeng Wang, Tao Tao, Jiali Zou, Zhu Qiu, Long Wang, Huimin Du, Ni Chen, Xuedong Yin

**Affiliations:** 1 Department of Breast and Thyroid, Guiyang Maternal and Child Health Care Hospital & Guiyang Children’s Hospital, Guiyang, China; 2 Department of Breast and Thyroid Surgery, Dongying People’s Hospital, Dongying City, Shandong China; 3 Key Laboratory of Molecular Oncology and Epigenetics, The First Affiliated Hospital of Chongqing Medical University, Chongqing, China; 4 Department of Breast and Thyroid Surgery, The First Affiliated Hospital of Chongqing Medical University, Chongqing, China; 5 Department of Oncology, The First Affiliated Hospital of Chongqing Medical University, Chongqing, China; 6 Department of Ultrasound, The First Affiliated Hospital of Chongqing Medical University, Chongqing, China

**Keywords:** Papillary thyroid carcinoma, Contrast-enhanced ultrasound, Lymph node metastasis

## Abstract

**Background:**

Contrast-enhanced ultrasound (CEUS) has been recently used for the assessment of cervical lymph node metastasis (LNM) to guide surgical operation in patients with papillary thyroid carcinoma (PTC). However, the specificity and sensitivity of CEUS reported from previous studies are not consistent. The objective of this study was to evaluate the diagnostic value of CEUS for the metastasis of cervical lymph nodes in PTC patients based on data from one regional central hospital.

**Methods:**

The diagnostic value of CEUS in preoperative LNM of PTC patients was concluded by comparing the results of CEUS on lymph node status with postoperative pathology examination. In addition, this study conducted hierarchical analysis of PTC patients to explore whether tumor size, different lymph node regions, and Hashimoto’s thyroiditis influence the assessment of CEUS.

**Results:**

This research study ultimately enrolled 965 PTC patients, including 266 males and 699 females with a mean age of 42.27 ± 11.34 years. A total of 527 patients were considered clinical-node negative, and 438 were clinical-node positive before surgery. The specificity, sensitivity, positive predictive value (PPV), negative predictive value (NPV) and accuracy of CEUS in the assessment of LNM in PTC patients were 56.00%, 71.00%, 57.06%, 69.76% and 62.59%, respectively. For central and lateral lymph nodes, the accuracy of CEUS in PTC patients was 49.43% and 54.30%, respectively. In addition, it was shown that the accuracy of CEUS in PTC patients with Hashimoto’s thyroiditis (HT) slightly decreased to 58.44%, and the accuracy of CEUS in PTC patients with non-HT in turn increased to 64.17%. The accuracy of CEUS in non-papillary thyroid microcarcinoma (PTMC) and PTMC patients was 65.68% and 61.24%, respectively. The accuracy of CEUS in predicting central LNM was significantly different between PTC patients with or without HT (P < 0.001) in this study but not for lateral lymph nodes (P = 0.114).

**Conclusion:**

The accuracy of CEUS in the assessment of LNM in PTC is not consistently satisfactory, especially for central lymph nodes, small tumor diameters, or patients with HT. More diagnostic technologies for abnormal lymph nodes should be considered in PTC patients.

**Supplementary Information:**

The online version contains supplementary material available at 10.1186/s12893-023-02199-w.

## Introduction

Papillary thyroid carcinoma (PTC) is the most common type of thyroid cancer, and its incidence has increased in recent years [1]. PTC is characterized by a predisposition of metastasis to cervical lymph nodes, and previous studies [2, 3] have shown that the incidence of lymph node metastasis (LNM) can reach 30-80%. In addition, LNM is often associated with local recurrence and distant metastases [4]. Therefore, complete dissection of suspicious abnormal lymph nodes is an important method to reduce the possibility of PTC recurrence.

Accurate preoperative assessment of cervical LNM is very important for patients with PTC. There are many technologies to evaluate cervical lymph nodes preoperatively, such as computed tomography (CT), magnetic resonance imaging (MRI), and ultrasonography (US). The identification of abnormal lymph nodes using the spatial resolution and contrast resolution of CT in PTC patients is not satisfactory or efficient [5]. Although MRI has some value in the assessment of lymph node metastasis, it is not currently available as a routine preoperative examination due to its high price. Ultrasound (US), which is recommended by the American Thyroid Association (ATA) [6] as the first choice for the diagnosis of thyroid cancer, has been recently used for the evaluation of cervical LNM in patients with PTC. Nevertheless, it is not reliable and accurate for the assessment of cervical LNM by US [4, 7]. Previous studies [8, 9] reported that only 20–31% of PTC patients with cervical LNM can be accurately detected by US.

In recent years, contrast-enhanced ultrasound (CEUS) has developed rapidly and has been used to assess and diagnose thyroid cancer. Zhang et al. [10] showed that CEUS had high specificity and contributed to the diagnosis of thyroid nodules. Moreover, previous studies reported that CEUS has high sensitivity and specificity for lymph node metastasis of PTC. A review [11] reported that CEUS emphasizes the microvascularization of the lymph nodes and improves ultrasound diagnostic accuracy for cervical.

lymph node staging after PTC diagnosis. However, the results from different regions are not consistent, and the application of CEUS in the evaluation of LNM is controversial. The objective of this study was to evaluate the diagnostic value of CEUS for the metastasis of cervical lymph nodes in patients with PTC.

## Materials and methods

### Patients

**Fig. 1:**
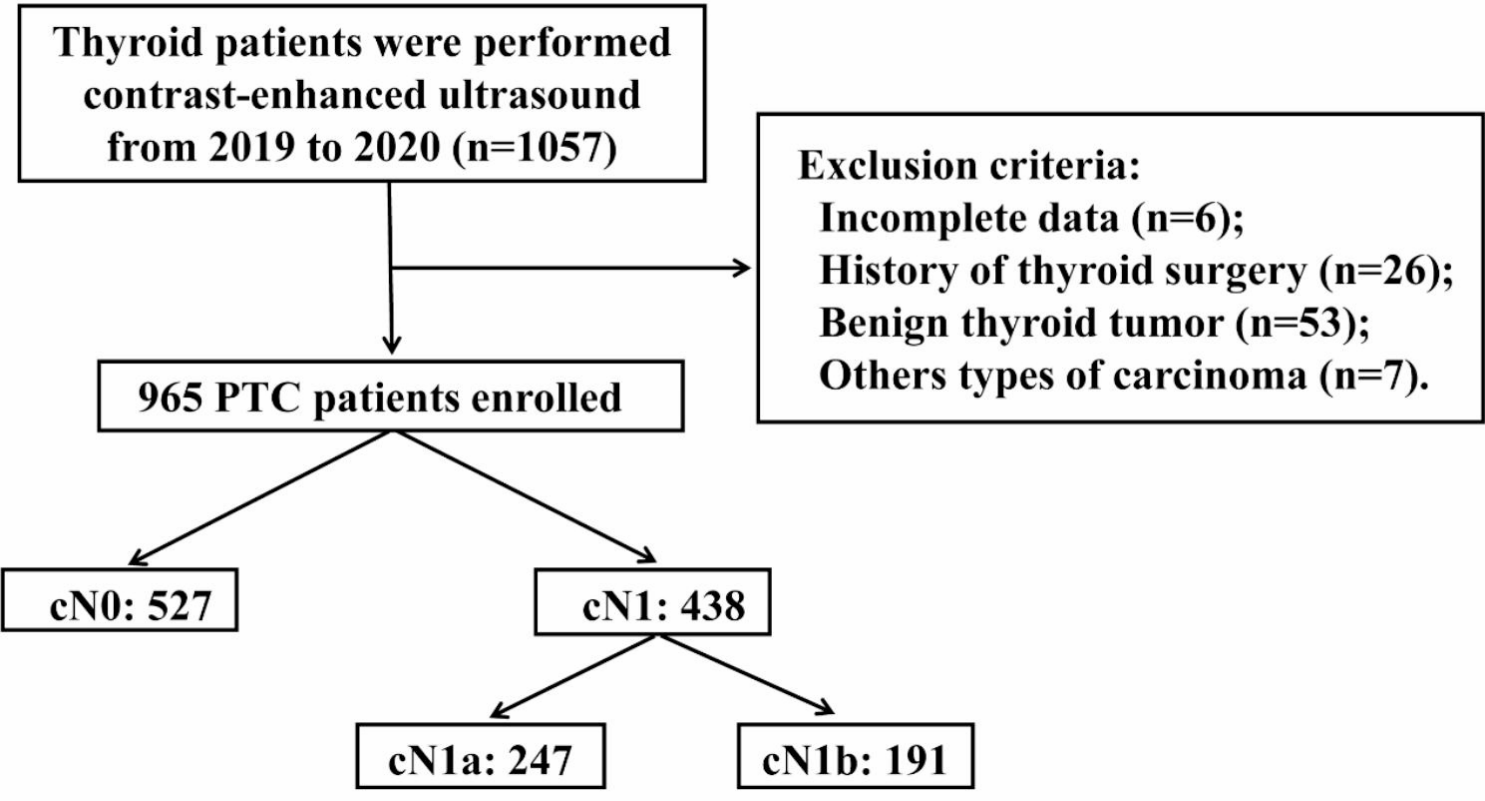
Flowchart of this retrospective study. PTC, papillary thyroid carcinoma; cN0, clinical-node negative; cN1, clinical-node positive; cN1a, clinical central node positive; cN1b, clinical lateral node positive

The clinical and pathological data of PTC patients who underwent CEUS before surgery were collected from January 2019 to January 2020. The exclusion criteria were as follows: (i) incomplete data, (ii) history of thyroid surgery, (iii) benign thyroid tumor and (iv) other types of carcinoma. A total of 965 patients were enrolled in this research study (Fig. 1). All PTC patients received thyroid lobectomy or total thyroidectomy combined with ipsilateral central lymph node dissection (CLND). Some patients received prophylactic or therapeutic lateral lymph node dissection (LLDN). Thyroid gland and lymph nodes that were removed during the operation were submitted for postoperative pathological examination. The results were independently diagnosed by two pathologists with more than 10 years of experience. If the result was in doubt, the diagnosis was made by a more experienced pathologist. The diagnostic value of CEUS in preoperative suspicious LNM of PTC patients was concluded by comparing the results of CEUS on lymph node status with postoperative pathology examination. In addition, hierarchical analysis was carried out to estimate the diagnostic value of CEUS in the subgroups as follows: (i) Hashimoto’s thyroiditis (HT) and non-Hashimoto’s thyroiditis (non-HT), (ii) papillary thyroid microcarcinoma (PTMC) and nonpapillary thyroid microcarcinoma (non-PTMC) and (iii) lymph nodes in different regions (central and lateral). In this study, we used histological diagnosis as the basis for diagnosing Hashimoto’s thyroiditis.

### Contrast-enhanced ultrasound (CEUS)

**Fig. 2:**
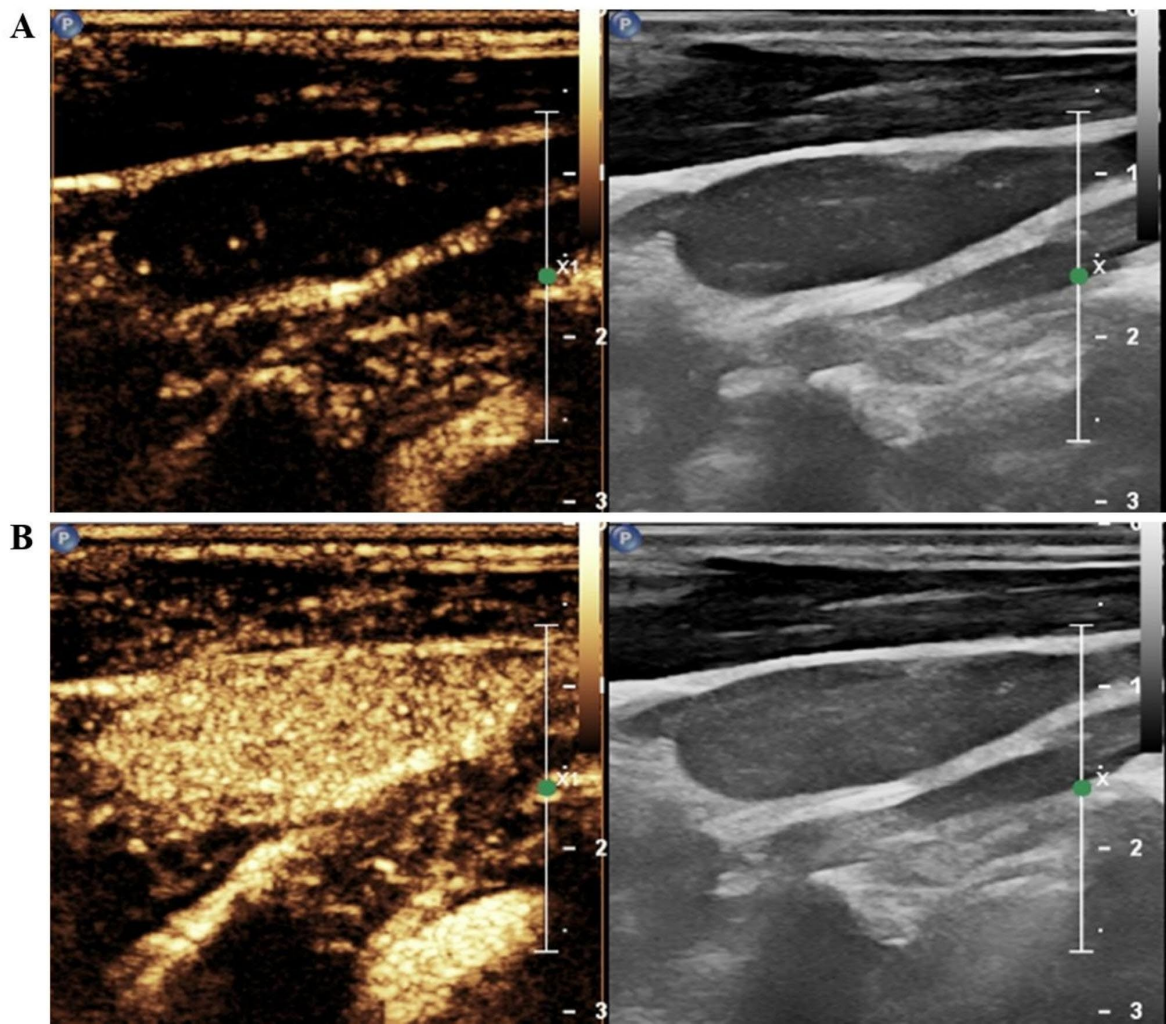
Metastatic lymph node. **(A)** Image of centrifugal perfusion of the contrast agent. **(B)** Image of inhomogeneously enhanced contrast agent

**Fig. 3:**
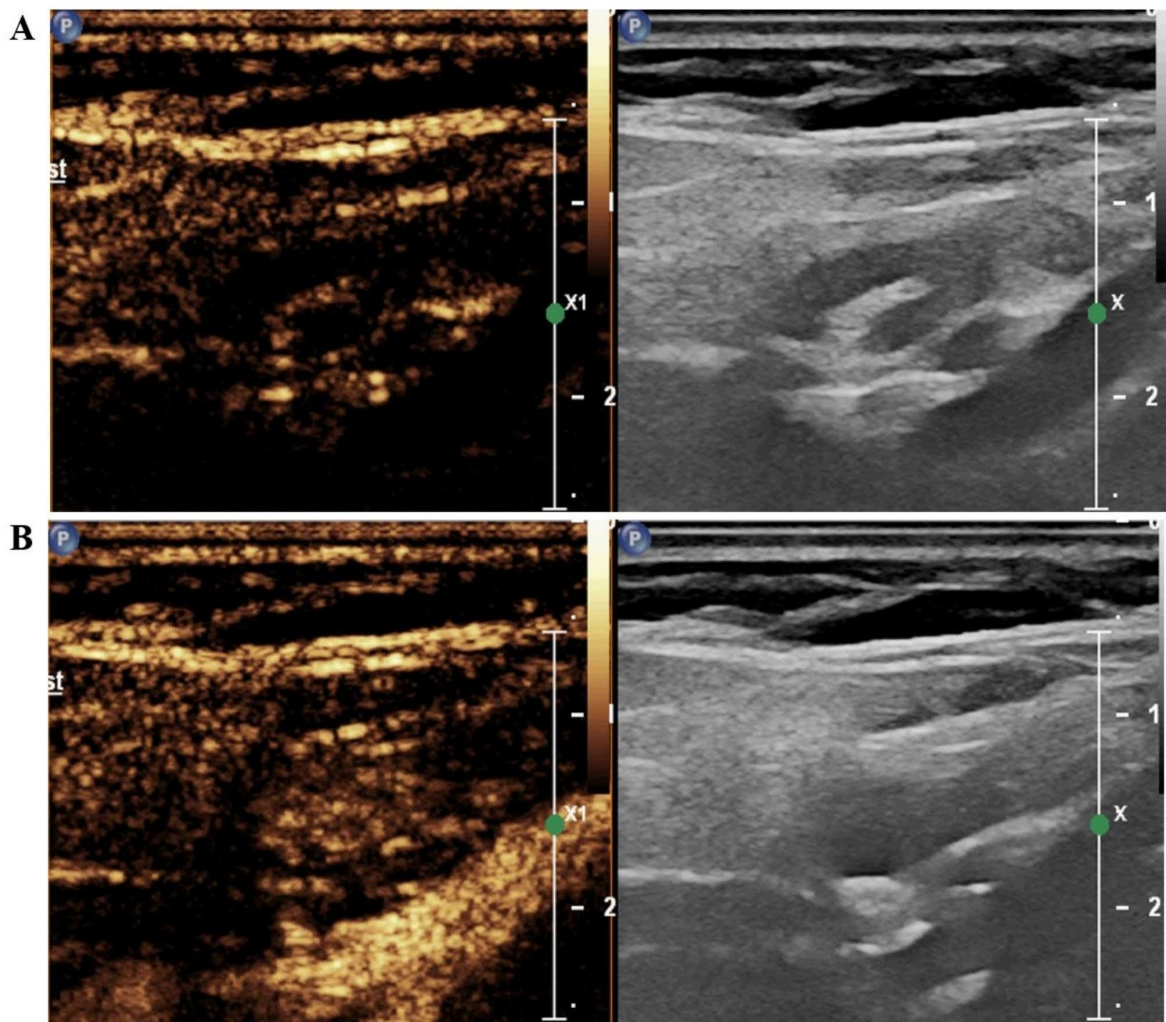
Reactive lymph node. **(A)** Image of centrifugal perfusion of the contrast agent. **(B)** The image of uniformly enhanced contrast agent

**Fig. 4:**
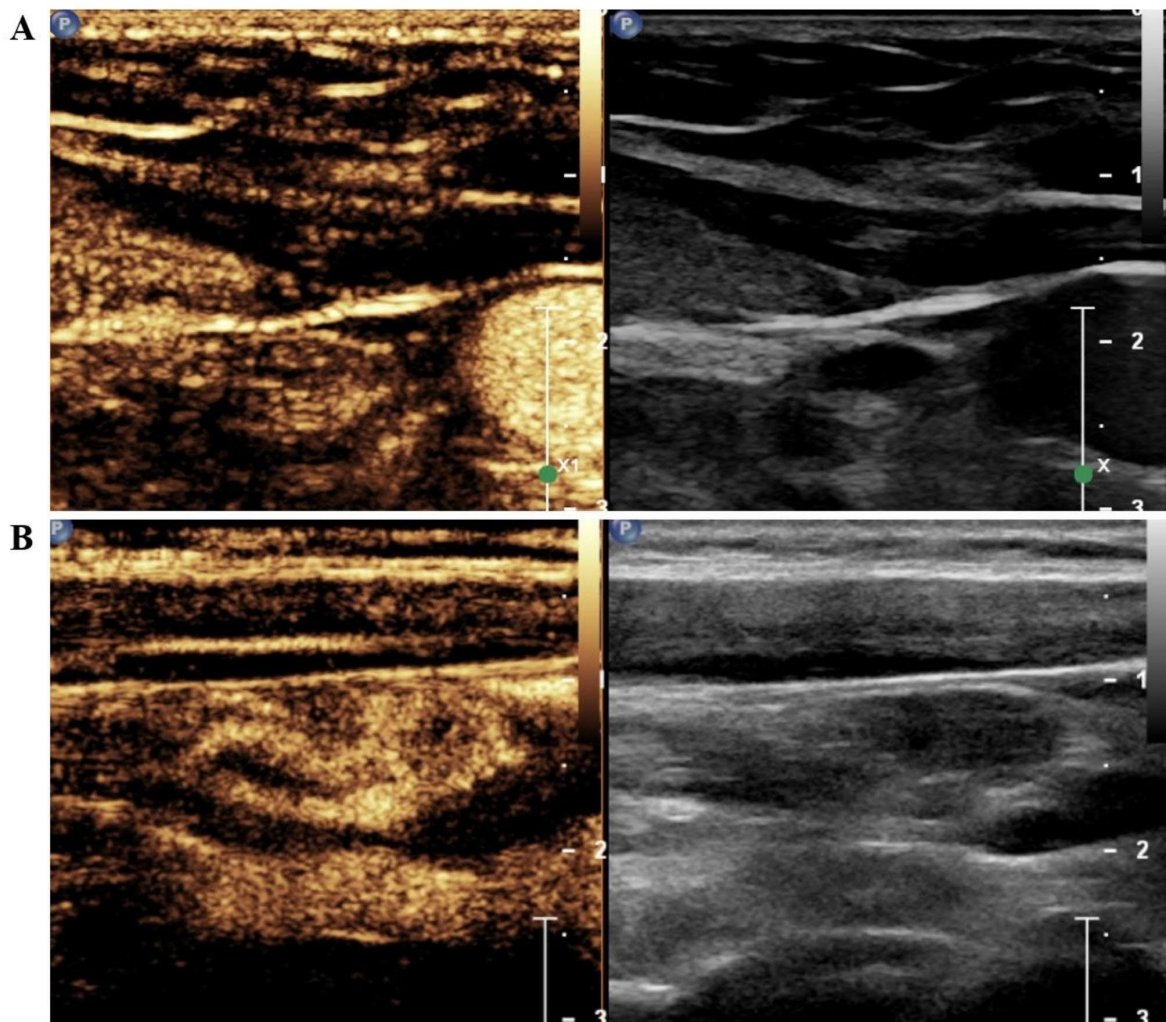
**(A)** Image of suspicious metastatic lymph nodes reported by contrast-enhanced ultrasound (CEUS) but no metastasis identified by pathological examination. **(B)** Image of reactive lymph nodes on contrast agent but with tumor metastasis identified by pathological examination

All individuals underwent preoperative CEUS. The contrast agent (SonoVue, Bracco, Italy) was mixed with 5 mL of saline, and 1.4–1.5 mL of the mixed suspension was rapidly administered into the patient’s peripheral vein. Then, images were collected at the time of injection of the contrast agent, and the time was not less than one minute. The imaging data obtained from CEUS were continuously stored. All individuals were monitored for adverse events until 20 min after the procedure. Finally, the images were discriminated by the sonographer. Based on the ultrasound features of LNM in thyroid cancer suggested by previous studies [8–10, 15], we used the following signs as a condition for diagnosing metastatic lymph nodes: centripetal perfusion and heterogeneous enhancement. The images show ultrasonogram sonography of clear metastatic lymph nodes (Fig. 2) and benign lymph nodes (Fig. 3) and images of lymph nodes with unclear diagnosis (Fig. 4). CEUS was performed by sonographers with at least 5 years of experience in the assessment of the thyroid nodule and its draining regional lymph nodes.

### Statistical analysis

T test, mean±standard deviation, chi-square, or Fisher’s exact test were employed to compare sex differences (male, female), age (< 55, ≥ 55 years), tumor size (≤ 10, <10 mm), bilaterality (yes, no), and Hashimoto’s thyroiditis (yes, no) in the PTC patients. Multivariate analysis was performed to explore factors linked with central lymph node metastasis in PTC patients. Specificity (= true negative/(false-positive + true negative)×100%), sensitivity (= true positive/(true positive + false-negative) ×100%), positive predictive value (PPV = true positive/(true positive + false-positive) × 110%), negative predictive value (NPV = true negative/(true positive + false-positive) × 110%) and accuracy were used to evaluate the value of CEUS in the diagnosis of LNM. SPSS version 22 software was employed for all analyses, and P < 0.05 was considered statistically significant.

## Results

### The clinical features of patients

In this study, 965 PTC patients were ultimately enrolled. These patients included 266 males and 699 females with a mean age of 42.27 ± 11.34 years. All the patients were evaluated by CEUS. A total of 527 patients were considered clinical-node negative (cN0), and 438 were clinical-node positive (cN1) before surgery. In this study, 627 (64.97%) had PTMC, and 231 (23.94%) had Hashimoto’s thyroiditis (HT), as identified by pathological examination. A total of 178 (18.45%) tumors presented bilaterality and 7 (0.73%) were located in the isthmus. The incidence of LNM in 965 PTC patients was 56.48% (545/956) (Table 1).

**Table 1:** Demographic and clinicopathologic characteristics of patients with PTC

	N=965	(%)
Sex		
male/female	266/699	27.56/72.44
Age	(42.27 ± 11.34)	-
<55 years/≥55 years	818/147	84.77/15.23
PTMC(≤10 mm)		
yes/no	627/338	64.97/33.03
Bilaterality		
yes/no	178/787	18.45/81.55
HT		
yes/no	231/734	23.94/76.06
Isthmus		
yes/no	7/958	0.73/99.27
cN1		
yes/no	438/527	45.39/54.61
LNM		
yes/no	545/420	56.48/43.52

PTC, papillary thyroid carcinoma; PTMC, papillary thyroid microcarcinoma; HT, Hashimoto’s thyroiditis; cN1, clinical-node positive; LNM, lymph node metastasis

### Risk factors for central lymph node metastasis

The percentage of central lymph node metastasis (CLNM) was 53.58% (517/965) in this article. The factors that are statistically related to CLNM may influence the assessment of CEUS on suspicious LNM before surgery. In the univariate analysis, the results revealed that male sex (p < 0.001), tumor size > 10 mm (p < 0.001) and bilaterality (p = 0.001) were significantly correlated with CLNM in PTC patients. However, age (p = 0.116) and HT (p = 0.472) were not correlated with CLNM. In addition, multivariate analysis showed that male sex (p < 0.001, OR: 1.792, 95% CI: 1.316–2.439), tumor size > 10 mm (p < 0.001, OR: 3.163, 95% CI: 2.370–4.222) and bilaterality (p = 0.011, OR: 1.585, 95% CI: 1.112–2.260) were independent risk factors for CLNM in PTC patients (Table 2).

**Table 2:** Univariate and multivariate analyses in CLNM PTC patients

	CLNM(+)(n,%)	CLNM(-)(n,%)	p	OR	95% CI	p
Male sex	171(64.29)	95(35.71)	<0.001	1.792	1.316–2.439	<0.001
≥ 55 years	70(47.62)	77(52.38)	0.116			
Tumor size(> 10 mm)	243(71.90)	95(28.11)	<0.001	3.163	2.370–4.222	<0.001
Bilaterality	116(65.17)	62(34.83)	0.001	1.585	1.112–2.260	0.011
HT	119(51.52)	112(48.48)	0.472			

PTC, papillary thyroid carcinoma; CLNM, central lymph node metastasis; HT, Hashimoto’s thyroiditis; OR, odds ratio; 95% CI, 95% confidence interval

### Diagnostic value of CEUS for lymph node metastasis

In this study, the specificity, sensitivity, PPV, NPV and accuracy of CEUS in the assessment of LNM in PTC patients were 56.00%, 71.00%, 57.06%, 69.76% and 62.59%, respectively. For central and lateral lymph nodes, the accuracy of CEUS in PTC patients was 49.43% and 54.30%, respectively (Table 3).

**Table 3:** Diagnostic accuracy of CEUS for LNM in PTC patients

	Specificity, %	Sensitivity, %	PPV, %	NPV, %	Accuracy, %
All patients(n = 965)	56.00	71.00	57.06	69.76	62.59
HT patients Yes(n = 231) No (n = 734)	51.69 56.62	62.68 75.34	67.42 54.00	46.46 77.26	58.44 64.17
PTMC patients Yes(n = 627) No (n = 338)	61.52 36.13	60.73 81.74	45.86 70.20	74.48 51.81	61.24 65.68
CLND patients(n = 965)	47.98	58.06	27.85	77.33	49.43
LLND patients(n = 407)	45.62	64.21	50.83	59.28	54.30

CEUS, contrast-enhanced ultrasound; LNM, lymph node metastasis; HT, Hashimoto’s thyroiditis; PTC, papillary thyroid carcinoma; PTMC, papillary thyroid microcarcinoma; CLND, central lymph node dissection; LLND, lateral lymph node dissection; PPV, positive predictive value; NPV, negative predictive value

To identify whether HT and anatomical division of the neck would affect the evaluation of CEUS on suspicious LNM, hierarchical analysis was performed. The results showed that the accuracy of CEUS in PTC patients with HT slightly decreased to 58.44%, and the accuracy of CEUS in PTC patients with non-HT in turn increased to 64.17% (Table 3). In 231 PTC patients with HT, for central and lateral lymph nodes, the accuracy of CEUS was 45.89% and 53.33%, respectively. In addition, in 734 PTC patients with non-HT, the accuracy of CEUS for central and lateral lymph nodes was 66.62% and 54.64%, respectively (Table 4).

**Table 4:** Diagnostic accuracy of central and lateral LNM with CEUS in PTC patients with or without HT

	Specificity, %	Sensitivity, %	PPV, %	NPV, %	Accuracy, %
HT patients					
+ CLND (n = 231) +LLND(n = 105)	45.45 52.63	46.60 66.67	34.45 53.73	58.04 52.63	45.89 53.33
Non-HT patients					
+ CLND (n = 734) +LLND(n = 302)	67.25 47.59	64.38 63.24	35.40 49.71	87.13 71.09	66.62 54.64

CEUS, contrast-enhanced ultrasound; LNM, lymph node metastasis; PTC, papillary thyroid carcinoma; HT, Hashimoto’s thyroiditis; CLND, central lymph node dissection; LLND, lateral lymph node dissection; PPV, positive predictive value; NPV, negative predictive value

### Diagnostic value of CEUS for PTMC and different LNM diameters

The accuracy of CEUS in non-PTMC and PTMC patients was 65.68% and 61.24%, respectively (Table 3). In addition, the metastatic lymph node diameter of these patients was selected and analyzed. The results showed that the larger the diameter of LNM was, the higher the sensitivity of CEUS in PTC patients (Table 5).

**Table 5:** Diagnostic sensitivity of different diameter ranges of LNM in PTC patients

Lymph node diameter(cN1)(mm)		Postoperative pathology		Total(%)	Sensitivity, %
		+			
> 0 and ≤ 5		36	23	59(13.47)	61.02
> 5 and ≤ 10		146	73	219(50)	66.67
> 10 and ≤ 15		62	26	88(20.09)	70.45
> 15 and ≤ 20		34	2	36(8.22)	94.44
> 20		34	2	36(8.22)	94.44
Total		312	126	438(100)	71.23

LNM, lymph node metastasis; PTC, papillary thyroid carcinoma; cN1, clinical-node positive

## Discussion

A clinical characteristic of PTC is the predisposition of metastasis to neck lymph nodes [12]. However, because of the small size and specific position, the assessment of metastatic lymph nodes by conventional US in patients with PTC is limited and unsatisfactory [7]. Previous studies have shown that the sensitivity of US for cervical LNM is less than 50% [13, 14]. CEUS is a new imaging modality that has high sensitivity for assessing the vascularity of tissue, enabling assessments of blood perfusion [15, 16], and it provides more useful information than conventional US. Previous experiences indicated that vascularity patterns and their changes are very important in distinguishing between benign and malignant thyroid nodes. CEUS is increasingly being used to assess lymph nodes in patients with PTC before surgery [17, 18].

Our study retrospectively analyzed CEUS counting in 965 patients with PTC, and the results showed that the accuracy of CEUS in the assessment of LNM in PTC patients was 62.80%. This result is significantly lower than that of previous studies [15, 19, 20] (Chen et al. 89.1%, Hong et al. 89.3%, Wei et al. 70.8%). A possible reason is that the analysis of previous studies was based on a very small population. Our study also indicated that the NPV of CEUS assessing lymph node metastasis in 965 individuals with PTC was 70.17%, which was also unexpected. The precise judgment of metastatic lymph nodes is very important to the surgical decision-making for patients with PTC. This precise treatment would favor reducing surgical trauma and tumor recurrence after surgery. The debate on which anatomical region of the cervical lymph node should be resected has not been resolved because of the lack of prospective clinical trials. For patients with cN0 disease, prophylactic lymphadenectomy is not recommended according to American and European guidelines [5, 21], and if the lymph node is considered to be metastatic before surgery, then therapeutic dissection of the lymph node is considered necessary. Preoperative diagnosis always determines individualized surgical treatment, and the thoroughness of the initial operation usually correlates with a lower incidence of tumor recurrence. According to the results of this study, the false-negative rate of lymph node metastasis of PTC evaluated by CEUS assessment is approximately 30%, which may lead to inadequate initial surgery for some patients and an increased local recurrence rate. To resolve this problem, frozen section examination (FSE) was suggested and could quickly assess the status of the neck lymph node during the process of surgery, which would help surgeons to better determine the scope of surgery (total thyroidectomy or thyroid lobectomy, ipsilateral central lymph node dissection or bilateral central lymph node dissection). Moreover, FSE could theoretically decrease the risk of recurrence. Raffaelli et al. [22] showed that FSE is able to change the extent of thyroidectomy in approximately one-fourth of PTC patients scheduled for thyroid lobectomy, and its overall accuracy can reach 90%. Unfortunately, due to the long time and high cost of FSEs, they have not been fully applied.

According to our analysis, the central lymph node is more difficult to evaluate than the lateral lymph node. Moreover, the central lymph nodes are the most likely areas for metastasis of PTC [12]. Central lymph node dissection is usually prone to serious complications, including injury to the recurrent laryngeal nerve, temporary or permanent dysfunction of the parathyroid and esophagotracheal leakage. It is important to know the status of lymph nodes before surgery. Previous studies have explained many factors associated with CLNM, such as tumor size >2 cm [23], age <45 years [24], capsule invasion [25], multifocality [25, 26], and male sex [27]. In our research, the clinical data of 965 patients with PTC were analyzed. The results showed that male sex, tumor size > 1 cm, and bilaterality served as independent risk factors associated with CLNM. It is suggested that more comprehensive preoperative evaluation should be considered for PTC patients when the conditions mentioned above are present.

HT is one of the factors that affects the accuracy of CEUS. HT is a common autoimmune thyroid disease that usually destroys thyroid cells through lymphocytic infiltration and interstitial fibrosis, eventually leading to hypothyroidism. The relationship between HT and lymph node metastasis of PTC has been controversial. A previous article [28] reported that patients with PTC coexisting with HT have higher aggressiveness. However, some authors [25, 29, 30] have demonstrated that PTC of HT has no relationship with tumor aggressiveness and is associated with lower rates of recurrence and longer overall survival. In addition, it might be a protective factor for lymph node metastasis in PTC [31]. Cappellacci et al. [32] revealed that HT was an independent risk factor for developing differentiated thyroid cancer (DTC); however, there was no difference in invasive features, such as extrathyroidal extension, vascular invasion, and nodal metastasis, between DTC patients with HT and non-HT. Our results were similar to those of Cappellacci, and there was no association between HT and lymph node metastasis in PTC.

Moreover, HT can also lead to inflammatory enlargement of regional lymph nodes, which increases the difficulty of preoperative evaluation of lymph node status in PTC patients by US [33, 34]. Few studies have investigated the predictive value of CEUS for the status of lymph nodes in PTC patients with coexistent HT. In our study, all patients were differentiated according to different clinicopathological features to explore the difference in the accuracy of CEUS. The accuracy (58.44% vs. 64.17%) and sensitivity (62.68% vs. 75.34%) of CEUS in assessing lymph nodes in patients with HT were significantly reduced compared with those in non-HT patients. Moreover, the accuracy (45.89% vs. 66.62%), specificity (45.45% vs. 67.25%) and sensitivity (46.60% vs. 64.38%) of CEUS on CLNM in patients with HT were significantly reduced compared with those in patients without HT. In addition, the accuracy of CEUS was significantly different in PTC patients with or without Hashimoto’s thyroiditis (P < 0.001). With respect to the evaluation of LLNM by CEUS, no significant difference was found (P = 0.114). The results suggested that the value of CEUS was significantly affected in the evaluation of lymph nodes in PTC patients with HT, especially in central lymph nodes.

Based on the previous literature and our knowledge, there are multiple possible reasons for the results above. i) The deep anatomical position of central lymph nodes makes the accuracy of assessment unsatisfactory. ii) Compared with other cancers, PTC is relatively inert, and lymph node metastasis is unobvious and small. iii) The central lymph node is close to the thyroid gland, and HT leads to inflammatory enlargement of local lymph nodes, which is not easy to distinguish from metastasis. These reasons may explain why CEUS is more accurate in assessing lymph nodes in the lateral region and in patients without HT.

## Conclusion

The precise evaluation of lymph node metastasis is the most important part of the preoperative examination for thyroid carcinoma. CEUS has been applied not only for the evaluation of thyroid nodules but also in the assessment of metastatic lymph nodes. However, the accuracy is not consistently satisfactory according to our study, especially for central lymph nodes in HT patients with small tumor diameters. Inaccurate assessment may further lead to unnecessary total thyroidectomy plus cervical lymph node dissection or incomplete dissection of lymph nodes. It is recommended that other techniques, such as contrast-enhanced CT/MR and intraoperative pathological biopsy of suspicious lymph nodes, be considered in addition to CEUS.

### Electronic supplementary material

Below is the link to the electronic supplementary material.

Supplementary Material 1

Supplementary Material 2

## Data Availability

All data generated or analyzed during this study are included in this published article and its supplementary file.
