# Supplementary material for: Diagnostic value of contrast-enhanced ultrasonography in the preoperative evaluation of lymph node metastasis in papillary thyroid carcinoma: a single-center retrospective study
Source: BMC Surg. 2023 Oct 24;23:325. doi: 10.1186/s12893-023-02199-w (PMC10599078; doi:10.1186/s12893-023-02199-w)
Supplement: Supplementary file 2 — Supplementary Material 2 [file 12893_2023_2199_MOESM2_ESM.docx]

The detail of the supplementary file：

1. The “Patients” column shows the patients involved in this study. All the name had been hidden.
2. The “gender” column shows the gender of the involved patients. “0” represents the female, and “1” represents the male.
3. The third column shows the age of patients involved in this study. “0” represents <55-years old, and “1” represents ≥55.
4. The fourth column shows the grade of each patient according to TI-RADS (Thyroid imaging reporting and data system).
5. The fifth column: “0” represents non-bilateral PTC, and “1” represents bilateral PTC.
6. The “PTMC” column: “0” represents the diameter of cancer is not more than 1cm, and “1” represents the diameter of cancer is more than 1cm .
7. The “hashimoto's thyroiditis” column: “0” represents that the hashimoto's thyroiditis disease had not been reported in the patient, and “1” represents the hashimoto's thyroiditis disease exists.
8. The “LNM” column: “0” represents that there was no cervical lymph node metastasis, and “1” represents that cervical lymph node metastasis had been diagnosed.
9. The “CLNM” column: “0” represents that there was no cervical lymph node metastasis, and “1” represents that cervical lymph node metastasis had been diagnosed.
10. The “lymph node dissection” column: “1” represents that only central lymph node dissection had been performed, and “2” represents that both central and lateral lymph node dissection had been performed.
11. The “LLNM” column: “1” represents that lateral lymph node dissection had been performed, “2” represents that lateral lymph node dissection had not been performed, and the blank space means that the data is not recorded.
12. The “twelfth to twenty-first” columns represent the different anatomical regions of the left cervical lymph nodes reported by the pathological system, and the “R2-R6” columns represent the different anatomical regions of the right cervical lymph nodes reported by the pathological system. In these columns, “0” represents that there is no lymph node metastasis according to the pathological report, “1” represents that lymph node metastasis had been detected in the pathological report, and “the blank space” means that the data is not recorded.
13. The “ceus:cn1” column: “1” represents that there was suspicious lymph node metastasis detected by contrast-enhanced ultrasonography, and “0” represents that there was no lymph node metastasis reported by contrast-enhanced ultrasonography.
14. The “region of LNM” column: “1” represents that there was only central LNM reported by contrast-enhanced ultrasonography, and “2” represents that that there was only lateral LNM reported by contrast-enhanced ultrasonography.
15. The “size of LNM（mm)” column represents the diameter of suspicious lymph node reported by ultrasonography: “1”: 0-5mm; “2”: 5-10mm; “3”: 10-15mm; “4”: 15-20mm; “5”: ≥20mm
16. The last columns from 25 to 32 represent the different anatomical regions of the suspicious cervical lymph node metastasis reported by contrast-enhanced ultrasonography. In these columns, “0” represents that there is no suspicious lymph node metastasis according to the CEUS, “1” represents that the suspicious lymph node metastasis had been reported by CEUS, and “the blank space” means that the data is not recorded.
